# Mechanisms of Firing Patterns in Fast-Spiking Cortical Interneurons

**DOI:** 10.1371/journal.pcbi.0030156

**Published:** 2007-08-10

**Authors:** David Golomb, Karnit Donner, Liron Shacham, Dan Shlosberg, Yael Amitai, David Hansel

**Affiliations:** 1 Department of Physiology, Ben-Gurion University, Be'er-Sheva, Israel; 2 Zlotowski Center for Neuroscience, Ben-Gurion University, Be'er-Sheva, Israel; 3 Laboratory of Neurophysics and Physiology, Université Paris René Descartes, Paris, France; 4 CNRS UMR 8119, Paris, France; 5 Franco-Israeli Laboratory of Neurophysics and System Physiology, Université Paris René Descartes, Paris, France; UFR Biomédicale de l'Université René Descartes, France

## Abstract

Cortical fast-spiking (FS) interneurons display highly variable electrophysiological properties. Their spike responses to step currents occur almost immediately following the step onset or after a substantial delay, during which subthreshold oscillations are frequently observed. Their firing patterns include high-frequency tonic firing and rhythmic or irregular bursting (stuttering). What is the origin of this variability? In the present paper, we hypothesize that it emerges naturally if one assumes a continuous distribution of properties in a small set of active channels. To test this hypothesis, we construct a minimal, single-compartment conductance-based model of FS cells that includes transient Na^+^, delayed-rectifier K^+^, and slowly inactivating d-type K^+^ conductances. The model is analyzed using nonlinear dynamical system theory. For small Na^+^ window current, the neuron exhibits high-frequency tonic firing. At current threshold, the spike response is almost instantaneous for small d-current conductance, *g*_d_, and it is delayed for larger *g*_d_. As *g*_d_ further increases, the neuron stutters. Noise substantially reduces the delay duration and induces subthreshold oscillations. In contrast, when the Na^+^ window current is large, the neuron always fires tonically. Near threshold, the firing rates are low, and the delay to firing is only weakly sensitive to noise; subthreshold oscillations are not observed. We propose that the variability in the response of cortical FS neurons is a consequence of heterogeneities in their *g*_d_ and in the strength of their Na^+^ window current. We predict the existence of two types of firing patterns in FS neurons, differing in the sensitivity of the delay duration to noise, in the minimal firing rate of the tonic discharge, and in the existence of subthreshold oscillations. We report experimental results from intracellular recordings supporting this prediction.

## Author Summary

About 25% of the neurons in the mammalian neocortex are inhibitory, namely reduce the activity of neurons they contact. These inhibitory neurons exhibit diversity of morphological, chemical, and biophysical properties, and their classification has recently been the focus of much debate. Even neurons belonging to a single class of “fast-spiking” (FS) display a large variety of firing patterns in response to standard square current pulses. Previous works proposed that this class is in fact a discrete set of neuronal subtypes with biophysical properties differing in a discontinuous way. In this work, we propose an alternative theory, according to which the biophysical properties of FS neurons are continuously distributed, but distinct firing patterns emerge due to highly nonlinear dynamics of these neurons. We ascertain this theory by exploring with mathematical techniques a biophysically based model of FS neurons. We demonstrate that variable firing responses of cortical FS neurons can be accounted for if one assumes heterogeneity in the strength of some of the ionic conductances underlying neuronal activity. Our theory predicts the existence of two main firing patterns of FS neurons. This prediction is verified by direct recordings in cortical slices.

## Introduction

Among inhibitory neurons in the neocortex, the “fast-spiking” (FS) compose the most prominent type. These neurons are characterized by brief action potentials with a width smaller than 0.5 ms followed by a deep monophasic afterhyperpolarization (AHP) [1,2]. Delayed rectifier currents of the types Kv3.1–Kv3.2 are responsible for these characteristics of FS action potentials [3,4]. The firing patterns of FS cells in response to a step of injected current are highly variable. Depending on the neuron and on the amplitude of the current pulse, FS cells fire action potentials immediately after the onset of the current step or after a prolonged delay, which can be on the order of several hundreds of milliseconds [2]. Interestingly, voltage-dependent subthreshold oscillations in the gamma range have been reported during the delay period. They typically occur in a narrow voltage range just negative to threshold [4,5]. The steady-state firing pattern is reached after an adapting [6], non-adapting, or accelerating [2] transient. This steady state can be tonic or bursting. In the latter case, the neuron fires rhythmic irregular bursts of action potentials; this activity pattern is named “stuttering” [7]. The goal of the present modeling study is to address the origin of this variability.

Bifurcation theory, which classifies how the behavior of dynamical systems changes as their parameters vary, reveals that in strongly nonlinear systems, qualitatively different dynamical regimes can emerge as a result of a continuous variation of some parameters [8]. Hence, heterogeneities in biophysical parameters of neurons can induce distinct classes of firing patterns even if their distributions are smooth. In the present paper, we propose that a great deal of FS electrophysiological variability is a consequence of heterogeneities in the maximal conductance of a slowly-inactivating d-type K^+^ current [9,10], known to be present in FS cells [11,12], and in the strength of the Na^+^ window current. This window current, governed by the overlap between the activation and inactivation curves of the Na^+^ current, affects the ability of the neuron to fire at low rates [13]. To assess this proposal, we consider a minimal, conductance-based neuronal model that incorporates these two ionic currents and a fast delayed rectifier K^+^ current. We investigate this model using techniques from nonlinear dynamical system theory. We find that as the conductance of the d-current and the overlap of the activation and the inactivation curves of the Na^+^ current are varied within a range compatible with experimental data [14], the model neuron displays a “”variability of firing patterns similar to those observed experimentally in FS cells.

Our study leads to several predictions that can be tested experimentally. In particular, we predict that FS cells that fire tonically at low rates do not exhibit subthreshold oscillations during the delay period. We present experimental results consistent with this prediction.

## Results

We study a minimal, single-compartment conductance-based model that incorporates a Na^+^ current, *I*_Na_, a fast delayed rectifier K^+^ current of the Kv3.1–Kv3.2 type, *I*_Kdr_, and a d-type K^+^ current, *I*_d_. The details of the model are given in the Model section of Materials and Methods.

### Variability in Firing Patterns in a Model of FS Neurons

**Figure 1:**
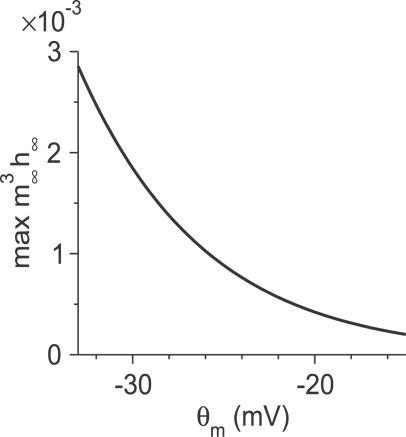
The Strength of the Na^+^ Window Conductance The maximal value of *m*_∞_^3^ *h*_∞_(V) with respect to *V* (Equation 6) is plotted as a function of θ_m_.

In all of this section, we assume that the system is noiseless. We will change the Na^+^ window current by varying the half-maximum potential, *θ*_m_, of the activation curve of *I*_Na_. As shown in Figure 1, the amplitude of the window current decreases as *θ*_m_ is more depolarized. The strength of the d-current is controlled by its maximal conductance *g*_d_. The amplitude of the current step injected into the neuron is denoted by *I*_app_.

For very large window current, i.e., small *θ*_m_ (*θ*_m_ < −31.4 mV for *g*_d_ = 0 and *θ*_m_ *<* −32.9 mV for *g*_d_ = 2 mS/cm^2^), the neuron is spontaneously active. In contrast, for very small window current, i.e., sufficiently large *θ*_m_ (*θ*_m_ > −15.2 mV for *g*_d_ = 0 and *θ*_m_ > −16.4 mV for *g*_d_ = 2 mS/cm^2^), the neuron remains quiescent for all amplitudes of the step current. In the intermediate range of *θ*_m_, the neuron is quiescent for *I*_app_ smaller than a threshold *I*_th_, while it fires spikes for *I*_app_ > *I*_th_. Then, depending on *θ*_m_, two qualitatively different behaviors of the neuron occur.

#### Firing patterns for small Na^+^ window current.

**Figure 2:**
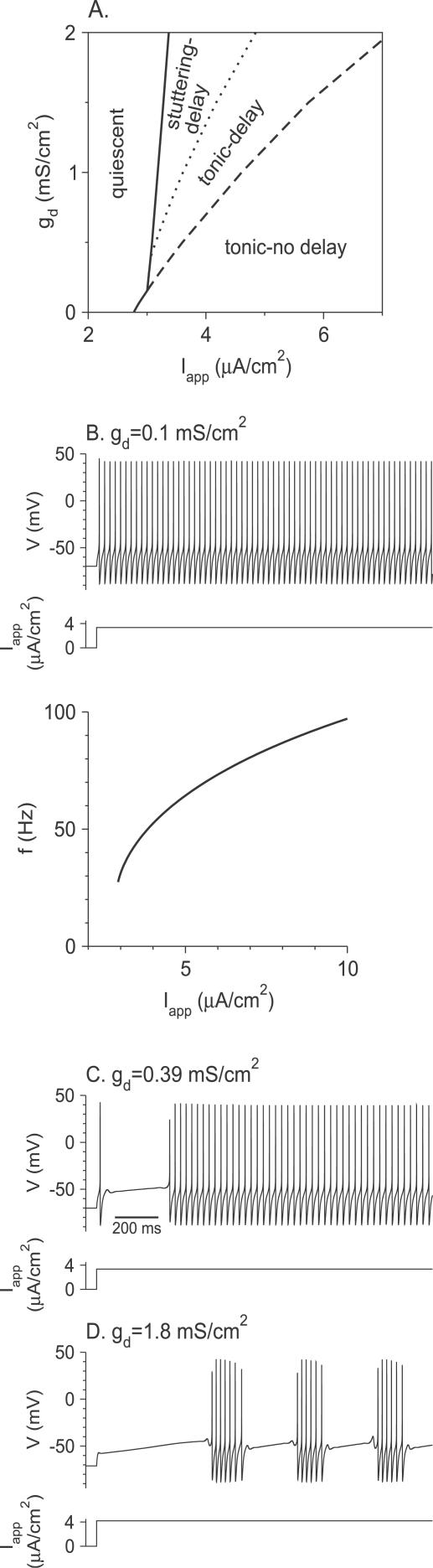
Responses of the Model Neuron to Noiseless Current Steps for Small Na^+^ Window Current (*θ*_m_ = −24 mV) (A) The phase diagram of the model neuron in the *I*_app_-*g*_d_ plane. The solid line represents the current threshold, *I*_th_, as a function of *g*_d_. To the left of this line, the neuron is quiescent. To the right of the dashed line, the spike response is almost immediate after the current step onset. Stuttering emerges on the dotted line via a torus bifurcation. (B) Top panel: the membrane potential *V* in response to a step of current for *g*_d_ = 0.1 mS/cm^2^ and *I*_app_ = 3.35 μA/cm^2^. The neuron exhibits non-delayed, high-frequency tonic firing. Bottom panel: the *f-I*_app_ curve for *g*_d_ = 0.1 mS/cm^2^ is discontinuous at the current threshold. The minimal frequency is 27.4 Hz. (C) For *g*_d_ = 0.39 mS/cm^2^, *I*_app_ = 3.35 μA/cm^2^, the neuron exhibits delayed, high-frequency tonic firing. (D) For *g*_d_ = 1.8 mS/cm^2^ and *I*_app_ = 4.2 μA/cm^2^, the neuron exhibits delayed stuttering.

The phase diagram of the different regimes of responses to a suprathreshold step of current as a function of the parameters *g*_d_ and *I*_app_ is shown in Figure 2A for *θ*_m_ = −24 mV. It is typical of the behavior of the model for small Na^+^ window current, and it contains four regions.

When *I*_app_ is small, in the region of the phase diagram marked “quiescent,” the neuron does not fire action potentials. The solid line that is the border of this region corresponds to the current threshold for firing, *I*_th_, which depends only weakly on *g*_d_.

In the region denoted “tonic-no delay,” the response of the neuron consists of a tonic discharge that begins immediately after the step onset. An example is shown in Figure 2B (top panel, *g*_d_ = 0.1 mS/cm^2^ and *I*_app_ = 3.35 μA/cm^2^). For sufficiently small values of *g*_d_ (*g*_d_ < 0.155 mS/cm^2^, intersection of the dashed line and the solid line), the “tonic-no delay” region extends down to the current threshold (solid line). At current threshold, the average firing frequency remains finite and varies discontinuously to zero. This is shown in Figure 2B (bottom panel) where we have plotted the *f*-*I*_app_ curve for g_d_ = 0.1 mS/cm^2^, for which the minimal firing frequency is 27.4 Hz. More generally, the firing frequency of the neuron is always substantially large in the entire “tonic-no delay” region.

In the “tonic-delay” region, the response of the neuron is still a tonic train of action potentials. In contrast to the previous case (“tonic-no delay” region); however, a substantial delay separates the discharge from the current step onset as shown in Figure 2C (g_d_ = 0.39 mS/cm^2^ and *I*_app_ = 3.35 μA/cm^2^). When 0.155 mS/cm^2^ < *g*_d_ < 0.393 mS/cm^2^, the left boundary of this region is the threshold line where the firing rate drops discontinuously to zero (unpublished data).

Finally, in the region located between the solid line and the dotted line, the neuron displays bursting, see Figure 2D (*g*_d_ = 1.8 mS/cm^2^ and *I*_app_ = 4.2 μA/cm^2^). As in the “tonic-delay” region, the discharge is delayed. The firing pattern that follows the delay consists of spikes occurring in clusters, similar to the *stuttering* described in a family of inhibitory FS interneurons [7]. This region is bounded to the left by the current threshold line. Detailed analysis shows that in the vicinity of this border, the interspike interval within the burst remains finite while the interburst duration diverges.

#### Firing patterns for large Na^+^ window current.

**Figure 3:**
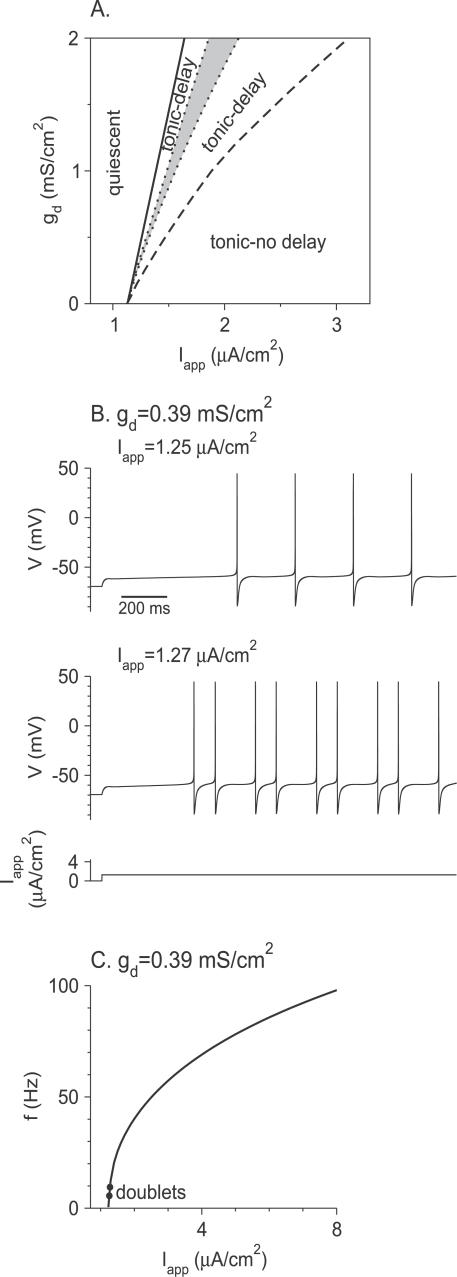
Responses of the Model Neuron to Noiseless Current Steps for Large Na^+^ Window Current (*θ*_m_ = −28 mV) (A) Phase diagram of the model neuron in the *I*_app_-*g*_d_ plane. Solid line: current threshold to action potential firing. Dashed line: to the right of the dashed line, the spike response is almost immediate after the current step onset. Period doubling bifurcations occur on the dotted lines, leading to doublet or complex firing (grey region). (B) Voltage traces for *g*_d_ = 0.39 mS/cm^2^ *.* Top: for *I*_app_ = 1.25 μA/cm^2^, the neuron displays low frequency delayed tonic firing. Bottom: for *I*_app_ = 1.27 μA/cm^2^, the neuron fires doublets of action potentials. (C) The steady-state *f*-*I*_app_ curve of the neuron; *g*_d_ is as in (B). The average firing frequency goes to zero at firing threshold. Between the two solid circles, the neuron fires doublets of spikes.

The phase diagram for *θ*_m_ = −28 mV is shown in Figure 3A. It is typical of the behavior of the model for large Na^+^ window current. Similar to the case of small window current, the firing discharge is delayed and periodic in a broad domain of the phase diagram. An example is shown in Figure 3B (top panel). However, a major difference is that now the steady-state frequency vanishes continuously at current threshold as can be observed in the *f-I*_app_ curve plotted in Figure 3C. This implies that the neuron is able to discharge at low rate, in contrast to what was found for small Na^+^ window current. For instance, in Figure 3B (top panel) the average rate is about 4 Hz. Another difference in the phase diagram is that a “stuttering” regime is no longer present. Finally, the “tonic-delay” region is divided into two components separated by a narrow domain where the discharge (gray region in Figure 3A), although still delayed, can display doublets [15], as shown in the panel at the bottom of Figure 3B, or more complex patterns (unpublished data).

#### Intermediate Na^+^ window current.

The phase diagram of the model remains qualitatively as in Figure 2A provided that *θ*_m_ is not too negative (*θ*_m_ > −25.5 mV). However, as *θ*_m_ becomes more hyperpolarized, the threshold *I*_th_ decreases and the minimal firing frequency becomes smaller. Delayed stuttering occurs for lower *g*_d_, but the range in current where it is observed becomes narrower and the number of spikes within a burst decreases. Eventually, for sufficiently hyperpolarized *θ*_m_, the *f*-*I*_app_ curve becomes continuous at threshold. Stuttering disappears in the sense that bursting behavior with distinct active and quiescent phases is no longer found. Instead, there is a regime where the neuron fires doublets of action potentials or has more complex firing patterns (for large values of *g*_d_). The detailed analysis of the mechanism by which stuttering disappears when *θ*_m_ becomes more hyperpolarized requires advanced bifurcation theory and is beyond the scope of the present paper.

#### The delay duration.

**Figure 4:**
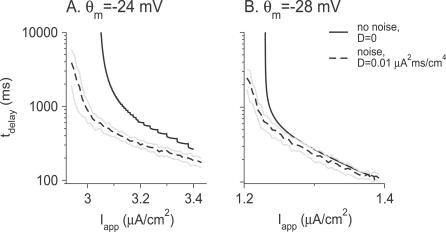
Dependence of the Delay Duration *t*_delay_ on the Amplitude of the Current Step *I*_app_ (A) *θ*_m_ = −24 mV. (B) *θ*_m_ = −28 mV. In the two panels, *g*_d_ = 0.39 mS/cm^2^. Solid lines: noiseless input (*D* = 0). Dashed lines: noisy input with variance *D* = 0.01 μA^2^ × ms/cm^4^. The delay duration was averaged over 50 trials. Gray lines represent one standard deviation around the mean value of *t*_delay_ for noisy input.

Figure 4A and 4B (solid lines) displays the dependency of the delay duration, *t*_delay_, on *I*_app_ for *θ*_m_ = −24 mV and *θ*_m_ = −28 mV, respectively (g_d_ = 0.39 mS/cm^2^). In both cases, a delay in the spike response occurs within a restricted range of *I*_app_. Its duration, which is a decreasing function of *I*_app_, goes to infinity at firing threshold and jumps discontinuously to zero when *I*_app_ becomes too large. Note that the divergence near threshold is sharper for *θ*_m_ = −28 mV than for *θ*_m_ = −24 mV. We will come back to this point later.

### The Roles of the Na^+^ Window Current and the Slow Inactivating K^+^ Current in Shaping the Neuron Discharge

In this section, we clarify the mechanisms underlying the different firing patterns described above and the dependency of the phase diagram on the Na^+^ window current. The time constant of the inactivation variable *b* of the d-current is *τ_b_* = 150 ms (see the Model section in Materials and Methods). Hence, *b* varies much more slowly than all the other dynamical variables of the model. The full dynamical system describing the neurons can subsequently be separated into fast (variables V, *h*, *n,* and *a*) and slow (variable *b*) subsystems. This allows us to dissect the dynamics of our model using the “fast–slow method” [13,16–18]. The first step in this method is to study how the attractors of the dynamics of the fast subsystem depend on the value of *b*, taken as a time-independent parameter. In a second step, one derives the dynamics of the full system taking into account the slow variations of *b.*

#### Dependency of the bifurcation structure of the fast subsystem on the Na^+^ window current.

**Figure 5:**
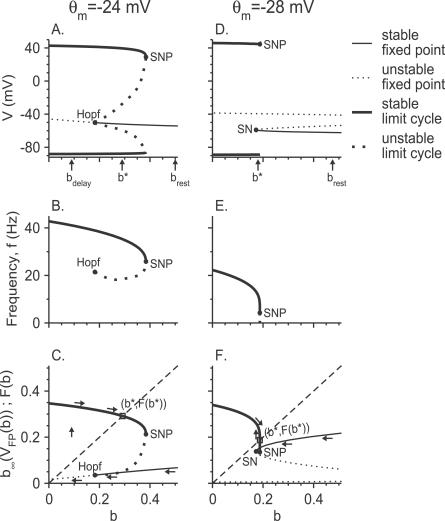
Bifurcation Diagrams and the Fast–Slow Analysis of the Model Neuron (A–C) are for small and (D–F) for large Na^+^ window currents. Parameters in (A–C) are as in Figure 2C: *θ*_m_ = −24 mV, *g*_d_ = 0.39 mS/cm^2^, *I*_app_ = 3.35 μA/cm^2^. Parameters in (D–F) are as in the top panel in Figure 3B: *θ*_m_ = −28 mV, *g*_d_ = 0.39 mS/cm^2^, *I*_app_ = 1.25 μA/cm^2^. (A,D) The bifurcation diagram of the fast subsystem in the *V*-*b* space. (B,E) The frequency *f* of the limit cycle of the fast subsystem, plotted as a function of *b* (*f*-*b* curves). (C,F) The functions *b*_∞_(*V*_FP_(*b*)) and *F*(*b*) (Equation 2) plotted as a function of *b*. Thin solid lines: stable fixed points; thin dotted lines: unstable fixed points; thick solid line: stable limit cycle (periodic state); thick dotted line: unstable limit cycle. Solid circles denote Hopf, saddle-node (SN), and saddle-node of periodics (SNP) bifurcation points. The value of *b* at rest (*I*_app_ = 0) is *b*_rest_, and b* is the value of *b* at steady state of the neuron. For *b*_delay_, see text. In (C,F), the intersection of the curve *b* = *F*(*b*) with the diagonal dashed line determines the value of b* (open square point). Arrows represent the evolution of the neuron from rest to its steady state following a current step injection of amplitude *I*_app_.

Consider the equation determining *V*_FP_, the value of the membrane potential at the fixed point of the fast subsystem. It is straightforward to see that when the window current is small (i.e., (*m*_∞_)^3^ *h*_∞_ is small in all the dynamic range of the voltage), *V*_FP_ decreases monotonously with *b* (Figure 5A) (see “The *b*-*V*_FP_ curve of the model neuron” in Materials and Methods for details). According to bifurcation theory, this implies that the fixed point of the dynamics of the fast subsystem can be destabilized as *b* decreases only via a Hopf bifurcation (a bifurcation where a limit cycle emerges) [18,19]. Whether the bifurcation actually occurs and the value, *b*_Hopf_, where it occurs, depend on *I*_app_ (*b*_Hopf_ = 0.18 for the parameters of Figure 5A–5C). As depicted in Figure 5A, the limit cycle that emerges at *b* = *b*_Hopf_ is unstable (subcritical Hopf bifurcation). At *b = b*_SNP_ (*b*_SNP_ > *b*_Hopf_), this unstable limit cycle coalesces in a “saddle–node of periodics” (SNP) bifurcation [8] with a stable limit cycle (*b*_SNP_ = 0.38 for the parameters of Figure 5A–5C). This limit cycle corresponds to tonic firing of action potentials in the fast subsystem, and it exists for *b* ≤ *b*_SNP_, both below and above *b* = *b*_Hopf_. In particular, for *b*_Hopf_ < *b* < *b*_SNP_, the fast subsystem is bistable since its fixed point is also stable in this range. The minimal and maximal values of the membrane potential on this limit cycle are plotted in Figure 5A as a function of *b*. Note that the frequency of the periodic motion on the limit cycles (the “firing rate” of the fast subsystem) decreases with *b* (Figure 5B).

In the case of large Na^+^ window current ((*m*_∞_)^3^ *h*_∞_, e.g., *θ*_m_ = −28 mV), the function *V*_FP_(*b*) is non-monotonous and the curve *V*_FP_(*b*), in the *b*-*V*_FP_ plane, displays a “Z shape” (Figure 5D and “The *b*-*V*_FP_ curve of the model neuron” section in Materials and Methods). The lower branch of this curve corresponds to a stable fixed point of the fast subsystem, whereas on the middle branch, the fixed point is unstable (saddle point). These two branches coalesce at some value *b* = *b*_SN_ (*b*_SN_ = 0.17 for the parameters of Figure 5D–5F), where the fast subsystem undergoes a saddle–node (SN) bifurcation [18]. For *b* < *b*_SN_, the dynamics of the fast subsystem display a stable limit cycle (Figure 5D and 5E) [8,18]. This stable limit cycle also exists in a small region for *b > b*_SN_, up to a value *b = b*_SNP_ where it disappears by coalescing with an unstable limit cycle (*b*_SNP_ = 0.187 for the parameters of Figure 5D–5F), i.e., via an SNP bifurcation. Hence, the fast subsystem displays bistability between a limit cycle and a stable fixed point in that small region of *b* values. The unstable limit cycle is homoclinic to the saddle.

#### The origin of the delayed spike response in the full system.

**Figure 6:**
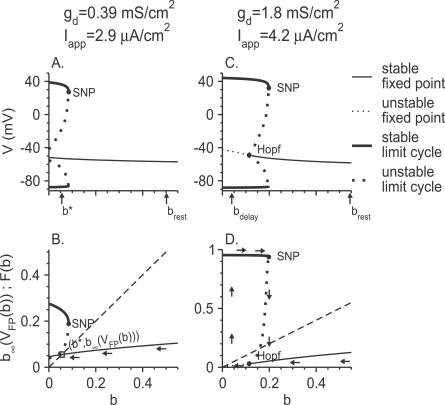
Fast–Slow Analysis of Neurons with Small Na^+^ Window Current Exhibiting a Depolarized Rest Potential or Stuttering Fast–slow analysis is described for *θ*_m_ = −24 mV. (A,B) Parameters are: *g*_d_ = 0.39 mS/cm^2^, *I*_app_ = 2.9 μA/cm^2^. (Except for *I*_app_, parameters are as in Figures 5A–5C and 2C. The neuron is quiescent at steady state.) (C,D) The parameters *g*_d_ = 1.8 mS/cm^2^ and *I*_app_ = 4.2 μA/cm^2^ are as in Figure 2D, and the neuron stutters at steady state. For each case, we plot the bifurcation diagrams of the fast subsystem in the *V*-*b* space (A,C), and the functions *b*_∞_(*V*_FP_(*b*)) for fixed points and *F*(*b*) for limit cycles (Equation 2) as functions of *b* (B,D). Symbols are as in Figure 5.

We now turn to the dynamics of the full system. Let us assume that the neuron is at rest (*I*_app_ = 0) and that the inactivation variable *b* is equal to *b*_rest_ (*b*_rest_ = 0.5 in the example of Figures 2C and 5A). At time *t* = 0, a current step is applied and *I*_app_ is raised abruptly (e.g., 3.35 μA/cm^2^ in Figure 5A–5C and 1.25 μA/cm^2^ in Figure 5D–5F). The evolution of the membrane potential of the neuron, *V,* right after the step onset is driven first by the dynamics of the fast subsystem with *b* ∼ *b*_rest_ (Figures 5A and 5D). The membrane depolarizes rapidly, the current *I*_d_ starts to inactivate, and the variable *b* decreases. During this process, which occurs on the slow time scale of *τ_b_*, the state of the neuron follows the fixed point of the fast subsystem adiabatically, for the applied current *I*_app_. In particular, the membrane potential of the neuron is *V*(*t*) ∼ *V*_FP_(*b*(*t*)), and the slow variable *b* continues to decrease as long as *b*_∞_(*V*_FP_(*b*(*t*))) < *b* (Equation 17). If the equation *b*_∞_(*V*_FP_(*b*)) = *b* has a solution, b*, for which the fixed point of the fast subsystem is stable, *b* stops evolving when it reaches that value. In that case, the state of the neuron converges to a stable fixed point where it does not fire action potentials. This situation, which happens when *I*_app_ is small, is depicted in Figure 6A and 6B. The value b* decreases with *I*_app_. The largest value of *I*_app_ for which the fixed point is stable is determined by the equation

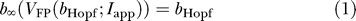
or by the equation *b*_∞_(*V*_FP_(*b*_SN_; *I*_app_)) = *b*_SN_ for small and large window *I*_Na_, respectively. For larger values of *I*_app_, a solution of the equation *b*_∞_(*V*_FP_(*b*)) = *b* exists only for a *b* value for which the fixed point of the fast subsystem is unstable (Figure 5C and 5F). As a result, *b* keeps decreasing until it crosses the bifurcation of the fast subsystem (*b = b*_Hopf_ or *b = b*_SN_). When this happens, *V* starts to diverge from *V*_FP_(*b*) and the neuron fires action potentials. The patterns of firing following the delay can also be assessed using the fast–slow analysis as we will explain below.

#### The properties of the delay period.

For small Na^+^ window currents, the fixed point of the fast subsystem that destabilizes at *b = b*_Hopf_ continues to exist in some range of values of *b* smaller than *b*_Hopf_. During the delay period, and before the fast subsystem crosses the Hopf bifurcation (*b > b*_Hopf_), the state of the neuron remains extremely close to this fixed point. Subsequently, it continues to track it for a while even after *b* has decayed below *b*_Hopf_, until it reaches a value *b*_delay_. Because of this “ramp effect” [13,20], the neuron starts to fire well after the inactivation variable has crossed *b*_Hopf_. This explains why in our simulations for *θ*_m_ = −24 mV (Figure 4A, solid line) *t*_delay_ is always significantly larger than the inactivation time constant of the d-current *τ_b_* (by at least a factor of 2).

In contrast, when the Na^+^ window current is large (e.g., *θ*_m_ = −28 mV), there is no “ramp effect,” since the fixed point of the fast subsystem does not exist for *b* < *b_SN_*. As a consequence, *t*_delay_ is on the order of *τ_b_* for most values of *I*_app_ (Figure 4B, solid line). It can be significantly larger than *τ_b_* only for *I*_app_ ≳ *I*_th_, where the dynamics of *b* itself becomes very slow.

The divergence of *t*_delay_ as *I*_app_ approaches the threshold *I*_th_ depends on the Na^+^ window current. As shown in the section “Dependence of the delay duration *t*_delay_ on *I*_app_ near current threshold for *D* = 0” in Materials and Methods, for *I*_app_ ≳ *I*_th_, *t*_delay_ diverges as (*I*_app_−*I*_th_)^1/2^ and −log(*I*_app_−*I*_th_) for small and large Na^+^ window currents, respectively.

#### The firing patterns after the delay period.

For small window current, the delay period ends when *b* has sufficiently decreased to a value *b*_delay_ < *b*_Hopf_ where the state of the neuron escapes from the fixed point of the fast subsystem. Once this happens, the state of the neuron converges rapidly toward the limit cycle of the fast subsystem with a value of *b* = *b*_delay_. Subsequently, the state of the neuron continues to evolve slowly, on a time scale of *τ_b_*, which is much larger than the time scale of the fast subsystem. Hence, at a given time, *t*, the state of the neuron is located very close to the limit cycle of the fast subsystem with a value of *b = b*(*t*). We denote this limit cycle by LC(*b*(*t*)). The state of the neuron follows LC(*b*(*t*)) many times before *b* changes significantly. Averaging the dynamics of *b* over a cycle, one finds that the evolution of *b* (Equation 17) can be approximated by:

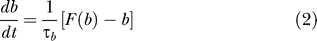
where F(*b*) is the time-average of the function *b*_∞_(*V*(*t*)) over LC(*b*) (see Figure 5C).

If *b*_delay_ *> F*(*b*_delay_)*, b* keeps *decreasing* below *b*_delay_ as the neuron fires action potentials. Eventually, it stabilizes at the value b* defined by *F*(b*) = b* (the solution of Equation 2 must exist in that case because in our model LC(*b*) exists up to *b = 0* and *F*(*b*) is always strictly positive). Therefore, the delay is followed by a transient during which the firing pattern displays reverse adaptation. Eventually, the neuron fires tonically and periodically.

In contrast, if *b*_delay_ *< F*(*b*_delay_), *b* starts to *increase* during the firing period. If the equation *F*(*b*) *= b* has a solution, b*, *b* eventually converges to b*. During this convergence, the neuron fires spikes with some frequency adaptation. Eventually, its state stabilizes on a limit cycle, LC(b*) corresponding to tonic periodic firing. Interestingly, if b* > b_Hopf_, LC(b*) coexists with a stable fixed point. This happens, for example, in the parameter set of Figure 5A. The highest value that b* may have is *b*_SNP_. Therefore, the smallest value of *I*_app_ for which b* exists is determined by the equation

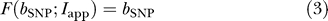
We consider the case where the value of *I*_app_ determined by Equation 3 is larger than the value of *I*_app_ determined by Equation 1. For *I*_app_ between those two values, the equation *F*(*b*) *= b* does not have a solution, but the state of the fast subsystem is on the limit cycle LC(*b*). In this case, *b* keeps increasing until it reaches its value at the SNP bifurcation, *b*_SNP_, beyond which there is no limit cycle (Figure 6C and 6D). At that point, the neuronal state jumps back to a stable fixed point, *b* decreases slowly again to *b*_delay_, and the whole process repeats itself. Therefore, in this case, the neuron displays “elliptic bursting” [13,18]). As a matter of fact, as shown in the section “The equation *F*(*b*) = *b* has no solution for large *g*_d_” in Materials and Methods, when *g*_d_ is sufficiently large, there is no solution to the equation *F*(*b*) *= b* in a broad region of *I*_app_. This explains the existence of a regime of bursting (stuttering) in the phase diagram (Figure 2A) for large *g*_d_ values. Note that the boundaries to the left and to the right of this region are given (in the limit of large *τ_b_*) by the equations *b*_∞_(*V*_FP_(*b*_Hopf_; *I*_app_)) = *b*_Hopf_ (Equation 1) and *F*(*b*_SNP_; *I*_app_) = *b*_SNP_ (Equation 3), respectively.

A similar analysis can be done when the Na^+^ window current is large and the delay ends at an SN bifurcation (when *b* crosses *b*_SN_). As in the case of small Na^+^ window current, two situations can be distinguished depending on whether *b*_SN_ is larger or smaller than F(*b*_SN_) (Figure 5F). In the first case, tonic firing occurs. Tonic firing also occurs in the second case unless the equation *F*(*b*) = *b* has no solution. In the latter situation, bursting is expected to occur. However, because the bistable region (*b*_SN_ < *b* < *b*_SNP_) is small and the period of the limit cycle is large in that region (Figure 5D and 5E), the fast–slow analysis correctly predicts the behavior of the full model only if τ_b_ is very large. As a matter of fact, for τ_b_ = 150 ms, we find doublets in place of well-defined bursting patterns.

#### Transient firing of spikes before the delay period.

As shown in Figure 2C, the neuron may fire a transient of a few (1–3) spikes before the delay period. A similar phenomenon is observed in experiments (i.e., [11], see results shown below). In our model, this behavior stems from the dynamics of *I*_Na_ and happens because the abrupt depolarization induces a rapid increase in the activation variable *m*, whereas the inactivation variable *h* decreases more slowly, on a time scale of a few ms. As a result, a rapid but transient increase in *I*_Na_ occurs, which may induce a few spikes before the neuronal dynamics converges to the new steady state of the fast subsystem. This effect resembles rebound excitation [13], although it happens even if there is no hyperpolarization before the injection of *I*_app_. It requires that the neuron be sufficiently depolarized by the current step. Note that the model neuron does not fire rebound spikes in response to release from hyperpolarizing current steps (Figure S1A).

### Effects of Noise on the Neuronal Dynamics

#### The effect of noise on the delay duration.

**Figure 7:**
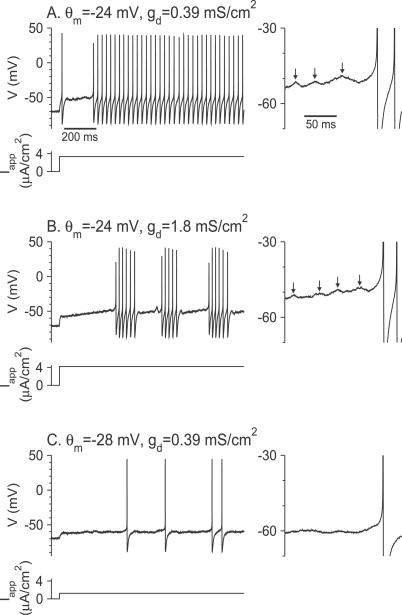
Voltage Traces and Firing Patterns of the Model Neuron in Response to a Noisy Current Step (A) Delayed tonic firing for *θ*_m_ = −24 mV, *g*_d_ = 0.39 mS/cm^2^, *I*_app_ = 3.35 μA/cm^2^. (B) Delayed stuttering for *θ*_m_ = −24 mV, *g*_d_ = 1.8 mS/cm^2^, *I*_app_ = 4.2 μA/cm^2^. (C) Delayed tonic firing for *θ*_m_ = −28 mV, *g*_d_ = 0.39 mS/cm^2^, *I*_app_ = 1.25 μA/cm^2^. In all three panels, the variance of the noise is *D* = 0.01 μA^2^ × ms/cm^4^. The time course of the mean applied current, *I*_app_, is also plotted. The membrane potentials during the delay periods are magnified in the panels on the right. Note the presence of subthreshold oscillations in (A) and (B). The peaks of these oscillations are denoted by the arrows.

The probability of firing is always non-zero in the presence of noise. Hence, strictly speaking, the very definition of a delay in firing is ambiguous. This probability, however, is (exponentially) small in the limit of small noise. In practice, delays can still be observed in a clear manner in the model unless the noise is large. This is shown for both small (Figure 7A and 7B) and large (Figure 7C) Na^+^ window currents.

**Figure 8:**
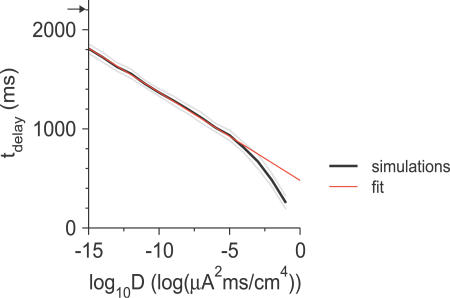
Dependence of the Averaged Delay Duration on the Noise Variance, *D* Black line: the delay duration, *t*_delay_, computed and averaged over 50 trials of the noisy external current step is plotted as a function of log(*D*). Parameters: *θ*_m_ = −24 mV (small window Na^+^ current), *g*_d_ = 0.39 mS/cm^2^, and *I*_app_ = 3.08 μA/cm^2^. Gray lines: one standard deviation confidence limit of the averaged *t*_delay_. Red line: fitting to the simulation results using the analytical prediction for small *D* (Equation 4). The arrow on the *y*-axis indicates the value of *t*_delay_ for a noiseless input (*D* = 0).

The delay duration, *t*_delay_, in the presence of noise with variance *D* = 0.01 μA^2^ ms/cm^4^ is plotted for small and large window *I*_Na_ in Figure 4A and 4B, respectively. Comparison with the noiseless case (solid line) shows that when the window *I*_Na_ is small, noise reduces *t*_delay_ dramatically. In contrast, for large window *I*_Na_, the effect of noise on the delay is weak, except for values of *I*_app_ in the vicinity of *I*_th_. This difference can be explained as follows. For small window current, the neuron state escapes slowly from the unstable fixed point of the fast subsystem during the delay period because of the “ramp effect.” This escape accelerates when noise increases. In the section “Dependence of *t*_delay_ on *D* for weak noise” in Materials and Methods, we derive the expression for *t*_delay_ as a function of *D* for low noise. One finds that:

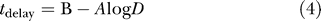
where *A* > 0 and *B* are constants (Equation 33). This equation provides a good fit to the results of our simulations over 11 orders of magnitude of *D* (Figure 8). Because of the logarithmic dependency, *t*_delay_ is considerably reduced in the presence of weak noise.

In contrast, when the window *I*_Na_ is large, there is no “ramp effect,” and *t*_delay_ is only weakly dependent on *D* (Figure 4B). An exceptional case is when *I*_app_ ≳ *I*_th_. In that case, the instability of the rest state of the full system is very weak and noise accelerates the escape from this rest state.

#### The spectrum of the membrane potential fluctuations during the delay period in the presence of noise.

**Figure 9:**
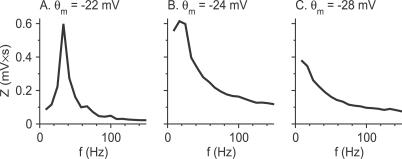
Fourier Spectrum of the Membrane Potential Fluctuations During the Delay Period in the Model for Three Different Strengths of the Na^+^ Window Current (A) *θ*_m_ = −22 mV, *g*_d_ = 0.8 mS/cm^2^, *I*_app_ = 4.9 μA/cm^2^; (B) *θ*_m_ = −24 mV, *g*_d_ = 0.39 mS/cm^2^, *I*_app_ = 3.23 μA/cm^2^; (C) *θ*_m_ = −28 mV, *g*_d_ = 0.39 mS/cm^2^, *I*_app_ = 1.25 μA/cm^2^.

When the Na^+^ window current is small, the fixed point of the fast subsystem near the bifurcation at *b = b*_Hopf_ is “spiral,” i.e., small perturbations around it decay or grow in an oscillatory manner when *b < b*_Hopf_ or *b > b*_Hopf_, respectively [8,21]. When noise is added, it excites this oscillatory mode, and therefore the membrane potential of the neuron displays sustained subthreshold oscillations (see Figures 7A and 7B and 9A and 9B). Similarly, oscillatory fluctuations induced by noise are observed during the interburst periods in the stuttering state.

In contrast, when the Na^+^ window current is large, the fixed point of the fast subsystem disappears in an SN bifurcation. Hence, in that case, small perturbations during the delay period decay exponentially and monotonously. As a consequence, the membrane potential does not display subthreshold oscillations during that period (Figures 7C and 9C).

#### Noise-induced stuttering for small Na^+^ window current.

**Figure 10:**
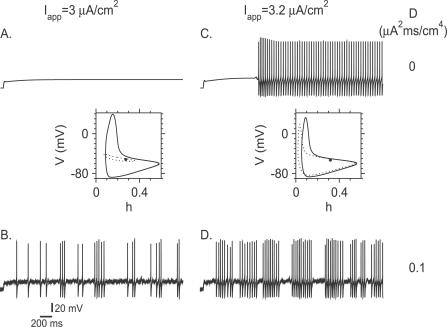
Noise-Induced Stuttering Activity for Small Na^+^ Window Current (*θ*_m_ = −24 mV) (A,B) Voltage traces in response to a step current of amplitude *I*_app_ = 3 μA/cm^2^. (A) In the absence of noise in the input (*D* = 0), the membrane potential depolarizes during the current step but the neuron does not fire action potentials. (B) Noise of variance *D* = 0.1 μA^2^ × ms/cm^4^ induces irregular bursts of action potentials (irregular stuttering). (C,D) Voltage traces in response to a step current of amplitude *I*_app_ = 3.2 μA/cm^2^. (C) For *D* = 0, the neuron exhibits delayed tonic firing. (D) For *D* = 0.1 μA^2^ × ms/cm^4^, the neuron stutters irregularly. For all panels, *g*_d_ = 0.5 mS/cm^2^. Projection of the phase portraits on the *V*-*h* plane for constant values of *b* (0.046 in (A) and 0.22 in (C)) are shown below the traces in (A,C). These values of *b* are either the fixed-point value (in (A)) or the average value on the limit cycle (in (C)). Solid circles denote stable fixed points, solid lines denote stable limit cycles, and dotted lines denote unstable limit cycles.

We have noted above that in the noiseless case, the stuttering region in the phase diagram is bounded between two lines defined by the equations *b*_∞_(*V*_FP_(*b*_Hopf_;*I*_app_)) = *b*_Hopf_ (Equation 1) and *F*(*b*_SNP_; *I*_app_) = *b*_SNP_ (Equation 3). On the left of the line determined by the equation *b*_∞_(*V*_FP_(*b*_Hopf_; *I*_app_)) = *b*_Hopf_, the rest state is the only stable attractor of the full system (Figure 10A). However, the stable limit cycle (LC(*b*)) is an attractor of the fast subsystem, in addition to the fixed point, as shown in the phase portrait in Figure 10A. Noise, which acts on a fast time scale, can induce random switching from the fixed point to this limit cycle before the slow dynamics of *b* enters into play. Once switching has happened, *b* starts to increase slowly, and the state of the neuron drifts on the limit cycle LC(*b*(*t*)), and therefore the neuron fires action potentials. The train of action potentials has some probability to stop if the noise switches the neuronal state back to the fixed point of the full system. If this has not happened before *b* has reached *b*_SNP_, firing stops, because at that point the limit cycle ceases to exist. In any case, this results in stochastic stuttering (Figure 10B).

Stochastic stuttering can emerge also for *I*_app_ larger than its critical value for which *F*(*b*_SNP_; *I*_app_) = *b*_SNP_. Then the tonic, periodic firing is the only stable attractor of the full system (Figure 10C), but the fast subsystem also has a stable fixed point (Figure 5A; phase portrait in Figure 10C). Noise can induce switching from the limit cycle to the fixed point. Once this has happened, *b* decreases either until the noise switches the state back to the limit cycle or until *b* reaches b_Hopf_, where the neuron switches back deterministically to the limit cycle of the fast subsystem (Figure 10D).

### Experimental Results

The minimal firing rates in the tonic discharge following the delay period is broadly distributed among FS neurons [22–24]. The theoretical results described above established correlations between the minimal firing rate of a neuron and the existence of subthreshold oscillations during the delay period. Neurons that fire at high rates display subthreshold oscillations during that period [22,23]. In contrast, neurons that can fire at low rates lack those oscillations. We report here experimental results supporting this prediction.

**Figure 11:**
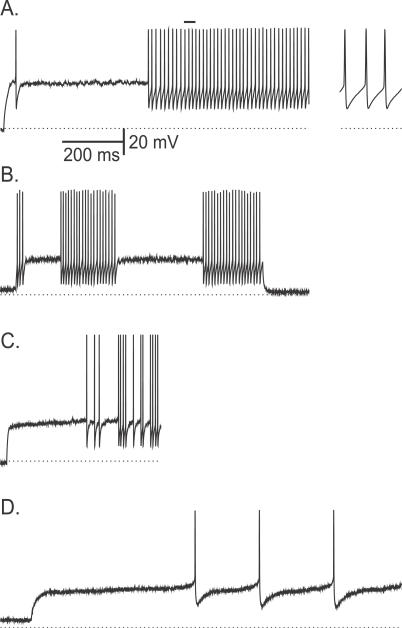
Experimental Results: Responses of FS Neurons to Just Suprathreshold Depolarizing Current Steps (A) An example of a neuron displaying high rate delayed tonic firing after a delay. The time interval below the top horizontal bar is magnified in the inset on the right. (B) A neuron exhibiting stuttering. (C) A neuron that exhibits delayed irregular firing. (D) A neuron that fires at low rates after a delay. The dotted line denotes −70 mV.

**Figure 12:**
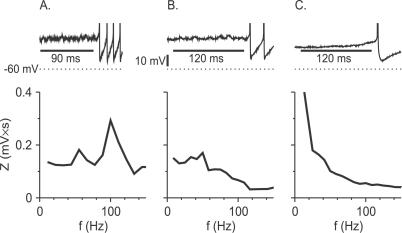
Experimental Results: Fourier Spectrum of the Membrane Potential Fluctuations during Delay Periods in FS Neurons Top: voltage time courses for three FS neurons exhibiting delayed firing. Bottom: the Fourier spectra of the subthreshold membrane potentials during the delay before spiking for these three neurons. (A) The minimal (just suprathreshold) firing frequency of this neuron is high (80 Hz). Pronounced subthreshold oscillations are observed during the delay period. (B) The minimal firing frequency of this neuron is lower than in (A) (38 Hz). The subthreshold oscillations exhibited by this neuron are less pronounced than in (A). (C) The minimal firing frequency of this neuron (4.3 Hz) is much lower than in (A) or (B). This neuron does not exhibit subthreshold oscillations during the delay period. Spectra were calculated over the time intervals denoted by the horizontal bars in the top panels.

The responses to steps of depolarizing current pulses were recorded intracellularly in FS neurons (*n* = 20, average ratio of the spike's rising phase *dV/dt* to falling phase *dV/dt* = 1.17 ± 0.29), as described in the section “Whole cell recordings and analysis” in Materials and Methods. Eight neurons responded to the depolarization onset almost instantaneously with a tonic non-adapting spike train. The other 60% of the neurons (*n* = 12) displayed a prolonged delay period, which was preceded by a transient firing of 1–3 spikes in some cases (Figure 11A and B), similar to the simulations of our model for low Na^+^ window current (Figure 7C). One cell from this group of 12 cells fired irregularly (Figure 11C). Eight out of those 12 cells had properties as reported in previous experimental studies of FS neurons (see also [22,25]). These eight cells exhibited a delay period followed by a tonic, high-frequency regular discharge (Figure 11A) or stuttering discharge with large, instantaneous intra-burst firing rate (Fig 11B). The minimal average firing frequencies in this group of neurons ranged from 25 to 220 Hz (average 81 ± 59 Hz) and the voltage threshold to action potential was −31.6 ± 5.0 mV. All the computed trial-average power spectra of the membrane fluctuations during the delay period (*n* = 4) displayed a peak within the gamma range (20–100 Hz). Two examples are shown in Figure 12A and 12B. These features are similar to those exhibited by our model neuron for small window current (Figure 9A and 9B).

Different properties were found in the remaining three neurons (out of these 12 cells) that displayed delay to firing. First, their minimal firing frequencies were under 10 Hz (average 6.8 ± 2.6 Hz) (Figure 11D), substantially lower than in the other group of eight neurons. Their spike threshold was −39.6 ± 6.4 mV, a significantly more hyperpolarized value than in the other group (*p* = 0.01, student *t*-test). These neurons did not exhibit stuttering behavior. Finally, spectral analysis of the fluctuations during the delay period failed to reveal subthreshold oscillations (Figure 12C). These properties are as predicted in our model for large Na^+^ window current (Figure 9C).

## Discussion

### Summary of the Theoretical Results

The minimal model of FS neurons studied in our work displays four types of behavior in response to a current step, depending on the Na^+^ window current and on the strength of the conductance of the K^+^ current *I*_d_.

1. When the Na^+^ window current and the conductance *g*_d_ are small, the neuron exhibits tonic, high-frequency firing that follows the current step onset almost immediately, even if the step amplitude is just above firing threshold.

2. When the Na^+^ window current is small and the conductance of the d-current is of intermediate strength, delayed high-frequency tonic firing occurs for just suprathreshold step amplitudes. The delay duration decreases as the step amplitude increases and abruptly jumps to zero at some critical value. Noise dramatically reduces this duration. Noise also induces subthreshold oscillations during the delay and can also induce stuttering.

3. For small Na^+^ window current but large values of the d-current conductance, the response to just suprathreshold input is delayed stuttering with high-frequency firing within the bursts. As the current step amplitude increases, the response becomes tonic firing, first delayed and subsequently and abruptly non-delayed. Other properties are as in 2.

4. For large value of the Na^+^ window current, the neuron responds with delayed tonic firing for small amplitude of the current step and non-delayed tonic firing when the amplitude of the current step is large. In contrast to what happens in 2, the average firing rate is low near firing threshold; noise very weakly affects the delay duration and does not induce subthreshold oscillations during the delay period.

### Na^+^ and K^+^ Currents in FS Cells and in Our Model

A large spectrum of K^+^ currents with different activation and inactivation properties and kinetics has been reported in FS neurons [7,11,26]. Delayed rectifier K^+^ channels from the Kv3.1–Kv3.2 types are responsible for the fast spike repolarization and strong AHP of these neurons [3,4,27]. Slowly inactivating K^+^ channels from the Kv1.1, Kv1.2, and Kv1.6 types have also been found in FS cells [12]; blockade of these currents with DTx-I eliminates delays to firing present in the control situation. The d-current incorporated in our model can be thought of as representing these slow channels.

We are aware of a single experimental study where the activation and inactivation properties of the Na^+^ channels in FS neurons were measured [14]. One conclusion of that study is that the overlap between the activation and inactivation curves of this current is small. Clearly this does not mean that the Na^+^ window current has no effect, since this will depend on the maximum conductance of this channel, a value which is not known. As a matter of fact, in our model, the inactivation mid-point potential, and the gain of the activation and inactivation functions are in accordance with the data provided by Martina and Jonas [14]. We take the same value of the Na^+^ conductance *g*_Na_ as in [3,28]. With this value, we find variability of firing patterns while varying the activation mid-point potential in a range compatible with the data provided by Martina and Jonas [14].

Throughout this article, we use the half-activation curve of *I*_Na_, *θ*_m_, to quantify the strength of the Na^+^ window current. Effects of modifying the window current by depolarizing or hyperpolarizing *θ*_h_ are similar to the effects of hyperpolarizing or depolarizing *θ*_m_, respectively (Figure S2). We did not include a persistent Na^+^ current in our minimal model because this current was not found in FS neurons [14]. However, if added to our model, this current would have an effect similar to increasing the Na^+^ window current (unpublished data).

Synchronization properties of neuronal networks are tightly related to single neuron properties [29–33]. For instance, increasing the strength of the Na^+^ window current transforms the bifurcation of the rest state from a Hopf type to an SN type. This change may switch an inhibitory-coupled network of FS neurons from an asynchronized state to a synchronized state [34]. Furthermore, Skinner et al. showed that networks of FS cells that possess both sufficiently strong Na^+^ window current (or persistent Na^+^ current) and *I*_d_, and that are coupled by both inhibitory and electrical coupling [35], may exhibit collective bursting oscillatory behavior [36]. Hence, the variability in single-cell properties, presented in this article, is very relevant to the network's behavior.

### Comparison of Model Behavior with Experimental Results

The firing patterns exhibited by our model include “classical” non-delayed tonic firing, delayed tonic firing, and delayed stuttering. These three patterns of firing are consistent with those described in recent experimental studies of FS cells (e.g., [7,22]) as well as in the experimental results reported in the present study. Increasing *I*_app_ in the model eventually causes the disappearance of the delay. This is also consistent with experimental observations [2].

A large variability is observed between FS neurons in their minimal firing rates in response to steady current. Whereas many FS cells have high steady-state minimal firing frequencies on the order of tens of Hz or more [2,23], the minimum firing rate of other FS neurons can be as low as 20 Hz [22] or even less than 10 Hz [37]. Especially, FS neurons with neurogliaform morphology can fire at low rates [24,38]. Although in our experimental data we classified neurons as FS based on their spike width and repolarization rate [1], and did not examine their morphology, our experimental results are consistent with such variability in the minimal firing rate. Further experimental work is needed to verify whether only FS neurons with neurogliaform morphology can fire at low rates. Relying on our modeling study, we propose that heterogeneities in the Na^+^ window current contribute strongly to variability in the minimal firing rate.

In our model, a delay in action potential firing is induced by a slow crossing of a bifurcation driven by the slowly inactivating d-current. Depending on the window current of the Na^+^ current, this bifurcation can be of Hopf or SN types. As a consequence, the properties of the neuron during the delay period depend also on the Na^+^ window current. In particular, subthreshold oscillations are found during that period (and also during the quiescent periods in stuttering patterns). This is consistent with the observation of subthreshold oscillations at frequencies in the gamma-range (20–100 Hz) in FS neurons in cortex [22] during delay or interburst periods. Moreover, in our model, these oscillations exist only when the *I*_Na_ window current is small (Figure 9A and 9B). Subsequently, we predict that subthreshold oscillations in an FS neuron are more likely to be observed in neurons with a large minimal firing frequency. Our experimental results (Figures 11 and 12) are consistent with this prediction.

Finally, noise can induce irregular stuttering in our FS model (Figure 10). Similar patterns were found in previous experiments (Figure 1C in [23]), as well as in the experiments reported here (Figure 11C).

### Some Limitations of Our FS Model

There are several physiological observations that the model does not replicate. The AHP and the spike amplitude are larger in the model (Figures 2 and 7) than in FS neurons recorded in slice experiments (Figure 11). Changing the reversal potential of the K^+^ current reduces the AHP of the model neuron to some extent. It may also happen that in FS cells, the spike-generating area is distant from the soma, and therefore action potentials recorded in the soma are filtered by cable properties. This effect, which cannot be included in our single compartment model, may contribute to the reduction of the AHP.

Another limitation of our model is that it can account neither for the substantial accommodation observed in some FS cells (AC cells in Figure 5 in [7]) nor for the burst of action potentials that precedes tonic firing sometimes observed in FS neurons (“b” cells in Figure 5 in [7]). Although our model may exhibit some adaptation, it is a result of the strong AHP, which causes *I*_d_ to inactivate during firing, but this inactivation is only weak. However, accommodation and initial bursting can probably be accounted for if one incorporates additional slowly activating K^+^ currents into the model.

Finally, stuttering FS cells that do not exhibit an initial delay in response to the injection of a step current are observed experimentally [6]. Such a behavior is not present in the phase diagram of Figure 2A. However, it can occur in the framework of our model if the reversal potential of the leak current is taken to be more depolarized (e.g., *V*_L_ = −60 mV in Figure S1B) than the reference parameter set *V*_L_ = −70 mV (Figure 1).

### Relation to Previous Theoretical Works and Models of FS Cells

To our knowledge, our work is the first to propose a minimal conductance–based model incorporating ionic channels known to exist in FS cortical interneurons and which accounts in a comprehensive way for the variability of firing patterns these cells display. The analysis we have made of this model builds on previous theoretical works. The role of the window *I*_Na_ in achieving low firing rates was considered in [13,18]. The fact that slowly inactivated K^+^ currents can induce delay to action potential firing and bursting was also described in [13,39]. The stuttering pattern displayed by our model is an example of “elliptic bursting” [18,20,40] (also named “SubHopf/Fold cycle” [19]). In addition, the present paper relates the appearance of subthreshold oscillations and the dependence of the delay duration on the levels of noise and applied current with the bifurcation structure of the fast subsystem.

Marder and colleagues proposed that for a specific pattern of activity, one can find parameter subspaces within which the model displays qualitatively, and even quantitatively, similar behavior [41,42]. They also proposed that neuronal function can be stabilized by homeostatic mechanisms ensuring that the neuron always remains in those subspaces [43]. Clearly, the bifurcation point cannot exist in such subspaces. There are, however, directions in parameter space along which the qualitative behavior of the neuron varies via bifurcations of the dynamics, as shown in our paper. These bifurcations can underlie the variability observed in the electrophysiological properties of FS cells.

### Predictions from the Model

We predict that for FS cells that exhibit delay before firing, the delay duration, *t*_delay_, decreases with the amplitude of the current step, *I*_app_, and disappears at a non-zero value as *I*_app_ is elevated (Figure 4A). When the neuron displays stuttering in response to just suprathreshold oscillations, we predict that elevating *I*_app_ will first increase the average number of spikes during the stuttering state (Figure 2D), and then will transform the cell into a tonic firing cell (Figure 2A). A depolarizing pre-pulse shortens *t*_delay_ and even eliminates it if the pre-pulse is large enough, but does not affect the stuttering behavior (Figure S1B). Similarly, a hyperpolarizing pre-pulse increases *t*_delay_. These predictions can be tested by current-clamp experiments.

Our theoretical work and the experimental results presented here suggest the existence in FS cells of two types of responses to step current pulses. They differ in the minimal firing frequencies, the properties of the membrane potential fluctuations during the delay period, and the sensitivity of the delay duration to noise. More specifically, we predict that the minimal firing rate, the sensitivity of *t*_delay_ to noise, and the presence of subthreshold oscillations of the membrane potential during the delay period are negatively correlated to the strength of the Na^+^ window current. Furthermore, we predict that FS neurons that can fire at low firing rates cannot stutter, and that increasing *g*_d_ artificially via dynamic clamp may convert a tonic-delay response into stuttering. These predictions can be tested in a detailed population study of electrophysiological properties of FS neurons.

The modeling results presented in this paper can be applied to understand the effect of some neuromodulators on the firing patterns of FS cells. Dopamine attenuates the d-type K^+^ current *I*_d_ in a subgroup of FS neurons [44]. Consistent with the results of our modeling study, dopamine also transforms the firing pattern of an FS cell from a tonic-delay type to the tonic-no delay type [44] (see also [45]). Na^+^ currents are affected by metabotropic glutamate receptor subtype 1 (mGluR1). As shown by [46], it shifts *θ*_h_ to more hyperpolarized potentials in pyramidal neurons, decreasing their Na^+^ window current. It also facilitates the persistent Na^+^ current *I*_NaP_ by shifting its activation curve leftward [46]. Similarly, serotonin makes *θ*_h_ more negative in pyramidal cells, and also reduces the maximal conductance of *I*_NaP_ [47]. If one assumes that these modulators have similar effects on Na^+^ currents in FS cells, our results suggest that they may modify qualitatively the firing patterns of these neurons.

## Materials and Methods

### Model.

Our model of FS cells is based on that of [3,28], with several modifications based on voltage clamp data. The current balance equation is

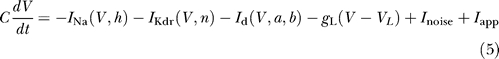
where *V* is the membrane potential of the neuron, *C* = 1μF/cm^2^ is the membrane capacitance, and the parameters of the leak current are *g*_L_ = 0.25 mS/cm^2^ and *V*_L_ = −70 mV. The external current injected into the neuron is denoted by *I*_app_.

The Na^+^ current *I*_Na_ is given by:

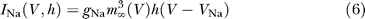
where the gating variables, *h* and *m*, follow:

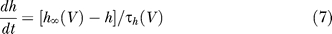


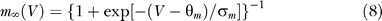


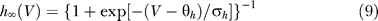


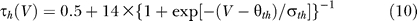
The parameters are: *g*_Na_ = 112.5 mS/cm^2^, *V*_Na_ = 50 mV, *σ_m_* = 11.5 mV, *θ_h_* = −58.3 mV, *σ_h_* = −6.7 mV, *θ_th_* = −60 mV, *σ_th_* = −12 mV [14]. In this work, we study the effect of the strength of the Na^+^ window current, controlled by the parameter *θ*_m_, on the dynamics of the neuron.

The delayed rectifier K^+^ current *I*_Kdr_ is of the Kv3.1–Kv3.2 type. It is responsible for the brief duration of the spike, about 0.5 ms [2,48], and for the high firing frequency [3,49]. It is given by:

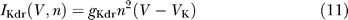
with:

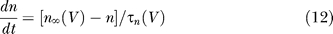


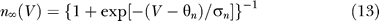


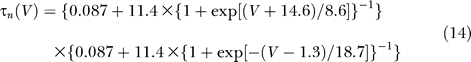
All the parameters of the delayed rectifier current are fixed: *g*_Kdr_ = 225 mS/cm^2^, *V*_K_ = −90 mV, *θ_n_* = −12.4 mV, *σ_n_* = 6.8 mV, *θ_tn_* = −27 mV, *σ_th_* = −15 mV [50].

The K^+^ current *I*_d_ incorporated in the model [10,11] has fast activation and slow inactivation. It is defined by:

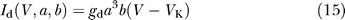


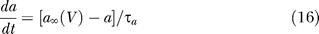


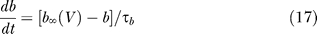


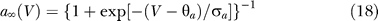


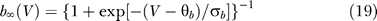
Throughout the paper, all the parameters of the d-current but *g*_d_ are fixed: *θ_a_* = −50 mV, *σ_a_* = 20 mV, *τ_a_* = 2 ms, *θ_b_* = −70 mV, *σ_b_* = −6 mV, *τ_b_* = 150 ms [51,52]. The parameter *g*_d_ is varied to study the effect of the strength of this current.

Finally, to study the effect of noise in the external input on the firing pattern of the neuron, we add an additional external input, *I*_noise_, of the form:

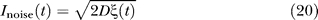
where *ξ*(*t*) is a Gaussian white noise with an average 0 and a unit variance, and *D* has the units of μA^2^ × ms/cm^4^ .

*Numerical methods.* Simulations were performed using the fourth-order Runge-Kutta method with a time step of 0.01 ms implemented as a C program or within the software package XPPAUT [53], which was used also for computing bifurcation diagrams.

*Delay.* The delay duration *t*_delay_ is defined to be the time from the onset of current injection, or, if the neuron fires transient 1–3 spikes, from the last transient spike to the first spike of the sustained firing. We define that the neuron shows a delay if *t*_delay_ is at least twice as large as the inter-spike interval during steady-state spiking *t*_ISI_, or if it is larger than both 100 ms and 1.2 *t*_ISI_.

*Fourier spectrum.* Discrete Fourier transforms of subthreshold oscillations were calculated numerically over a time window of *T*_FT_ ending *T*_BS_ = 5 ms before the first spike of the steady-state firing. The absolute values of the Fourier components were averaged over *n*_R_ repetitions of the same stimulus. Parameters for Figure 9 are: *T*_FT_ = 120 ms, *n*_R_ = 20. Parameters for Figure 12 are: *T*_FT_ = 90 ms, *n*_R_ = 5 (A), *T*_FT_ = 120 ms, *n*_R_ = 13 (B), *T*_FT_ = 120 ms, *n*_R_ = 11 (C).

### The *b*-*V*_FP_ curve of the model neuron.

The bifurcations of the fast subsystem when *b* varies depend on the shape of the function *V*_FP_(*b*), where *V*_FP_ is the value of the membrane potential of the neuron at the fixed point of the dynamics for fixed *b*. Equivalently, one can relate the bifurcations to the shape of the curve *b* = *b*(*V*_FP_), in the *b*-*V*_FP_ plane (the “*b*-*V*_FP_ curve”), which is defined by (see Equations 5, 6, 11, and 15):

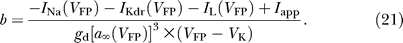
where

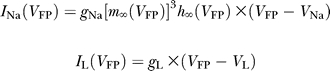
and

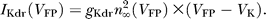
The denominator in Equation 21 is positive and increases with *V*_FP_. The numerator therefore is positive in the relevant range of *V*_FP_ for which *b* > 0. The functions *I*_L_(*V*_FP_) and *I*_Kdr_(*V*_FP_) are increasing with *V*_FP_. Only the function *I*_Na_(*V*_FP_) may decrease with *V*_FP_. Therefore, for a small Na^+^ window current, *b* decreases monotonously with *V*_FP_. This happens for instance for *θ_m_* = −24 mV (Figure 5A). In contrast, if the overlap between the activation and the inactivation curves of the Na^+^ current is sufficiently large [13], the term −*I*_Na_(*V*_FP_) in Equation 21 can contribute substantially to make the function *b*(*V*_FP_) be non-monotonous. This happens for instance for *θ_m_* = −28 mV (Figure 5D).

### Dependence of the delay duration *t*_delay_ on *I*_app_ near current threshold for *D* = 0.

We estimate *t*_delay_, the duration of the delay to firing of action potentials, using the “fast–slow method,” and derive the dependence of *t*_delay_ on *I*_app_ near the current threshold *I*_th_ in the noiseless case. During the delay period, the fast subsystem is at its fixed point, and *b* decreases slowly. We use this fact to compute the scaling of the divergence of *t*_delay_ with *I*_app_−*I*_th_ for *I*_app_ ≳ *I*_th_ for the two bifurcation scenarios.

*Hopf bifurcation of the fast subsystem.* The evolution of *b* is given by Equation 17, and it becomes very slow when *b* approaches *b*_∞_(*V*). For large *τ_b_*, *V* follows the curve *V*_FP_(*b*) during the delay period (Figure 5A). We denote by *bx̃* the solution of the equation 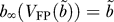 , and define *Vx̃* ≡*V*_FP_( *bx̃*) Note that the fixed point of the fast subsystem for *b* = *bx̃* is unstable if the neuron fires following the delay. When *I*_app_ is near *I*_th_, *V*_FP_(*b*) is approximated by *Vx̃* [13], and therefore 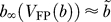 Equation 17 for the evolution of *b* is approximated by

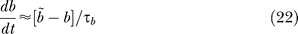
Using Equation 22 for computing *t*_delay_ is justified because *t*_delay_ is determined mainly by the slow dynamics of *b* when it is near *bx̃*. Before the current step is applied at time *t* = 0, the system is at rest with *b* = *b*_rest_. The subsystem converges immediately to its fixed point on the slow time scale, and the solution to Equation 22 is

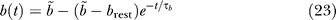
According to Equation 3.13a from [20], *t*_delay_ is determined by the equation

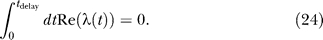
Near a Hopf bifurcation,

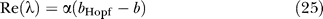
where *b*_Hopf_ is the value of *b* at the Hopf bifurcation and *α* is a constant. Substituting Equation 23 in Equation 24 and using Equation 25, we obtain

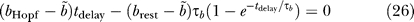
Near the threshold current *I*_th_, *t*_delay_ >> τ_b_, and Equation 26 becomes

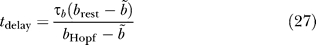
Generically, when *I*_app_−*I*_th_ is small, *bx̃* depends only weakly on *I*_app_ and *b*_Hopf_ − *bx̃* depends linearly on *I*_app_−*I*_th_. Therefore, *t*_delay_ scales as (*I*_app_−*I*_th_) ^−1^.

*Saddle-node bifurcation of the fast subsystem.* The dynamics are very slow near the SN bifurcation, occurring at (*b*_SN_, *V*_SN_). Neglecting the changes in *V*_FP_ during the evolution, Equation 23 becomes

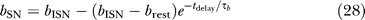
where *b*_ISN_ = *b*_∞_(*V*_SN_). Namely,

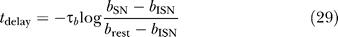
Generically, when *I*_app_−*I*_th_ is small, *b*_SN_−*b*_ISN_ depends linearly on *I*_app_−*I*_th_. Therefore, *t*_delay_ scales as −log(*I*_app_−*I*_th_).

### Dependence of *t*_delay_ on *D* for weak noise.

We calculate the dependence of *t*_delay_ on the noise variance, *D*, for weak noise and weak window *I*_Na_. In this case, the delay ends because the fast subsystem is destabilized via a Hopf bifurcation. According to Theorem 4.1 in [40], assuming that the variance of the noise, *D*, is neither too large nor too small, *t*_delay_ is determined by the equation

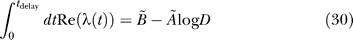
where *Ax̃* and *Bx̃* are constants. As above, *t*_delay_ is determined mainly by the slow evolution near *bx̃*, and therefore one can use the approximation *V*_FP_(*b*) ≈ *Vx̃*. Substituting Equations 23 and 25 in Equation 30, we obtain

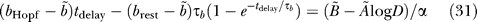
Solving this equation for *t*_delay_, in the limit *t*_delay_ >> *τ*_b_, we obtain

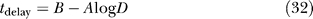
where 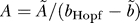 and 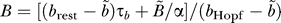 .

### The equation *F*(*b*) = b has no solution for large *g*_d_.

We consider the case that, for a certain value of *g*_d_, *g*_d_ = *g*_d1_, and a step current with an amplitude *I*_app_, there is a solution to the equation *F*(*b*) = *b*. We prove here that for a large enough *g*_d_, a solution to this equation does not exist for this value of *I*_app_. The function *F*(*b*) is defined only when the limit cycle exists, namely only for *b* ≤ *b*_SNP_. Since the current *I*_d_ depends on *g*_d_ and *b* only through the product *g*_d_ *b* (Equation 15), *b*_SNP_ for any other value of *g*_d_ is

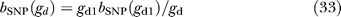
This means that *b*_SNP_(*g*_d_) is very small for large *g*_d_.

We continue by noticing that: (1) *F*(*b*) is the time-average of the function *b*_∞_(*V*(*t*)) over LC(*b*) (Equation 2); (2) *b*_∞_(*V*) is a positive, decreasing function of *V* (Equation 19); and (3) *V* ≤ *V*_Na_. Therefore, *F*(*b*) ≥ *F*(*b*_∞_(*V*_Na_)). From the fact that *b*_SNP_(*g*_d_) decreases with *g*_d_ (Equation 33), one finds that, for large enough *g*_d_, *F*(*b*_∞_(*V*_Na_)) > *b*_SNP_(*g*_d_). Since *F*(*b*) is defined only for *b* ≤ *b*_SNP_(*g*_d_), we obtain that *F*(*b*) > *b,* and there is no solution to the equation *F*(*b*) = *b* if *g*_d_ is large enough. Therefore, the full system cannot exhibit a tonic firing state. If the rest state of the neuron is unstable, the neuron stutters. In practice, *F*(*b*) is much larger than *F*(*b*_∞_(*V*_Na_)) because the membrane potential spends a large fraction of its period in subthreshold values, and therefore *g*_d_ should not be extremely large to prevent a solution of the equation *F*(*b*) = *b*.

### Whole-cell recordings and analysis.

Mice (CD1, 21–28 d old) were deeply anaesthetized with pentobarbital, decapitated, and their brains quickly removed into cold (5 °C) physiological solution. Coronal cortical slices (400 μm thick) were cut with a vibratome (Campden Instruments, http://www.campdeninstruments.com) and then transferred to a holding chamber where they were kept at room temperature for at least 1 h before recording, continuously bubbled with 95% O_2_, 5% CO_2_. Recording was done in a chamber mounted on an upright microscope equipped with IR/DIC optics (Nikon physiostation EC-600), where they were held at 32–34 °C and constantly perfused. The normal bathing solution contained (in mM): 124 NaCl, 3.5 KCl, 2 MgSO_4_, 1.25 NaHPO_4_, 2 CaCl_2_, 26 NaHCO_3_ and 10 dextrose, and was saturated with 95% O_2_, 5% CO_2_ (pH 7.4). Whole-cell recordings were made from neurons in the barrel field. Patch recording micropipettes (4–6 MΩ) were filled with a solution containing (in mM) 125 K gluconate, 5 NaCl, 2 MgCl_2_, 10 EGTA, 10 HEPES, and 2 Na_2_-ATP, pH 7.2, 280 mOsm). Voltages were recorded with a patch clamp amplifier (AxoPatch 2B, Axon Instruments, http://www.axon.com), and digitally sampled at 10 kHz. Data acquisition and analysis were performed with Labview (National Instruments, http://www.ni.com). Series resistance was typically <15 MΩ. During all recordings, 50 μM DL-2-amino-5-phosphopentanoic acid (AP5, Sigma, http://wwwsigmaaldrich.com) and 6,7-dinitroquinoxaline-2,3-dione (DNQX; 20 μM, Sigma) were present in the bath to block excitatory transmission.

*Identification of FS neurons.* Non-pyramidal neurons were targeted by their soma and proximal dendrites image under the IR/DIC microscope. Among those, FS neurons were identified according to their electrophysiological properties. A neuron was classified as an FS neuron if: 1) it fired brief spikes with fast, deep, monophasic AHPs [1]; and 2) the ratio of the spike's rising phase *dV/dt* to falling phase *dV/dt* was smaller than 2. Previous studies revealed that the morphological correlate of FS neurons can be either “basket” [7] or “neurogliaform” [24,54,55]. Most FS neurons express parvalbumin [56], but others express somatostatin [57]. Thus, this type of interneuron may be heterogeneous in terms of its morphology or chemical content, but we did not use these criteria for our classification.

## Supporting Information

Figure S1 Voltage Traces of the Responses of the Model Neuron to Current Steps (A) The neuron does not fire rebound spikes in response to release from hyperpolarizing current steps. Parameters: *θ*_m_ = −24 mV, *g*_d_ = 0.39 mS/cm^2^, *I*_app_ = 0, *I*_app_ of the hyperpolarizing current step is −8 μA/cm^2^, *V*_L_ = −60 mV.
(B) The neuron exhibits stuttering without delay for weak window *I*_Na_ and depolarized V_L_, that is raised to −60 mV from the reference parameter set of −70 mV. Parameters: *θ*_m_ = −24 mV, *g*_d_ = 1.8 mS/cm^2^, *I*_app_ = 1.7 μA/cm^2^.
(68 KB PDF)

Figure S2 Response of the Model Neuron to Noiseless Current Steps for Modified *θ*_h_ Values (A) Parameters: *θ*_m_ = −24 mV, *θ*_h_ = −54.3 mV, *g*_d_ = 0.39 mS/cm^2^. Left: voltage trace for *I*_app_ = 2.38 μA/cm^2^. Right: the steady-state *f*–*I*_app_ curve of the neuron. The average firing frequency goes to zero at firing threshold.
(B) Parameters: *θ*_m_ = −28 mV, *θ*_h_ = −62.3 mV, *g*_d_ = 0.39 mS/cm^2^. Left: voltage trace for *I*_app_ = 1.95 μA/cm^2^. Right: the steady-state *f*–*I*_app_ curve is discontinuous at the current threshold. The minimal frequency is 23.3 Hz.
(87 KB PDF)

## Abbreviations

AHP: afterhyperpolarization
FS: fast-spiking
SN: saddle–node
SNP: saddle–node of periodics
