# Supplementary material for: Mechanisms of Firing Patterns in Fast-Spiking Cortical Interneurons
Source: PLoS Comput Biol. 2007 Aug 10;3(8):e156. doi: 10.1371/journal.pcbi.0030156 (PMC1941757; doi:10.1371/journal.pcbi.0030156)
Supplement: Supplementary file 1 [file pcbi.0030156.sg001.pdf]

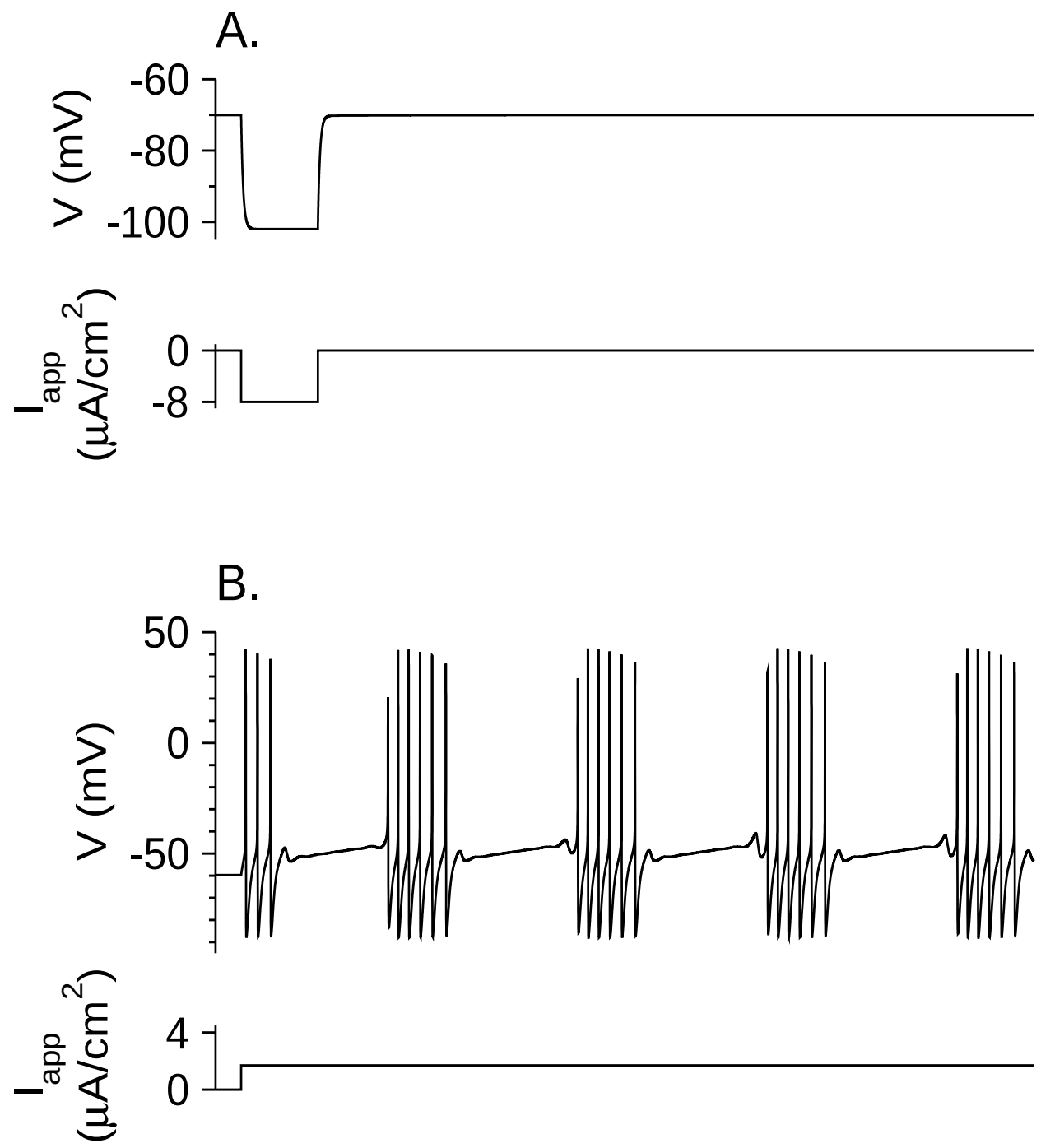

Figure S1. Voltage traces of the responses of the model neuron to current steps.

A. The neuron does not fire rebound spikes in response to release from hyperpolarizing current steps. Parameters: $\theta_{\mathrm{m}} = -24\,\mathrm{mV}$, $g_{\mathrm{d}} = 0.39\,\mathrm{mS/cm^2}$, $I_{\mathrm{app}} = 0$, I_{app} of the hyperpolarizing current step is -8 $\mu\mathrm{A/cm^2}$, $V_{\mathrm{L}} = -60\,\mathrm{mV}$. B. The neuron exhibits stuttering without delay for weak window I_{Na} and depolarized V_{L}, that is raised to -60 mV from the reference parameter set of -70 mV. Parameters: $\theta_{\mathrm{m}} = -24\,\mathrm{mV}$, $g_{\mathrm{d}} = 1.8\,\mathrm{mS/cm^2}$, $I_{\mathrm{app}} = 1.7\,\mu\mathrm{A/cm^2}$.
