# Supplementary material for: Mechanisms of Firing Patterns in Fast-Spiking Cortical Interneurons
Source: PLoS Comput Biol. 2007 Aug 10;3(8):e156. doi: 10.1371/journal.pcbi.0030156 (PMC1941757; doi:10.1371/journal.pcbi.0030156)
Supplement: Supplementary file 2 [file pcbi.0030156.sg002.pdf]

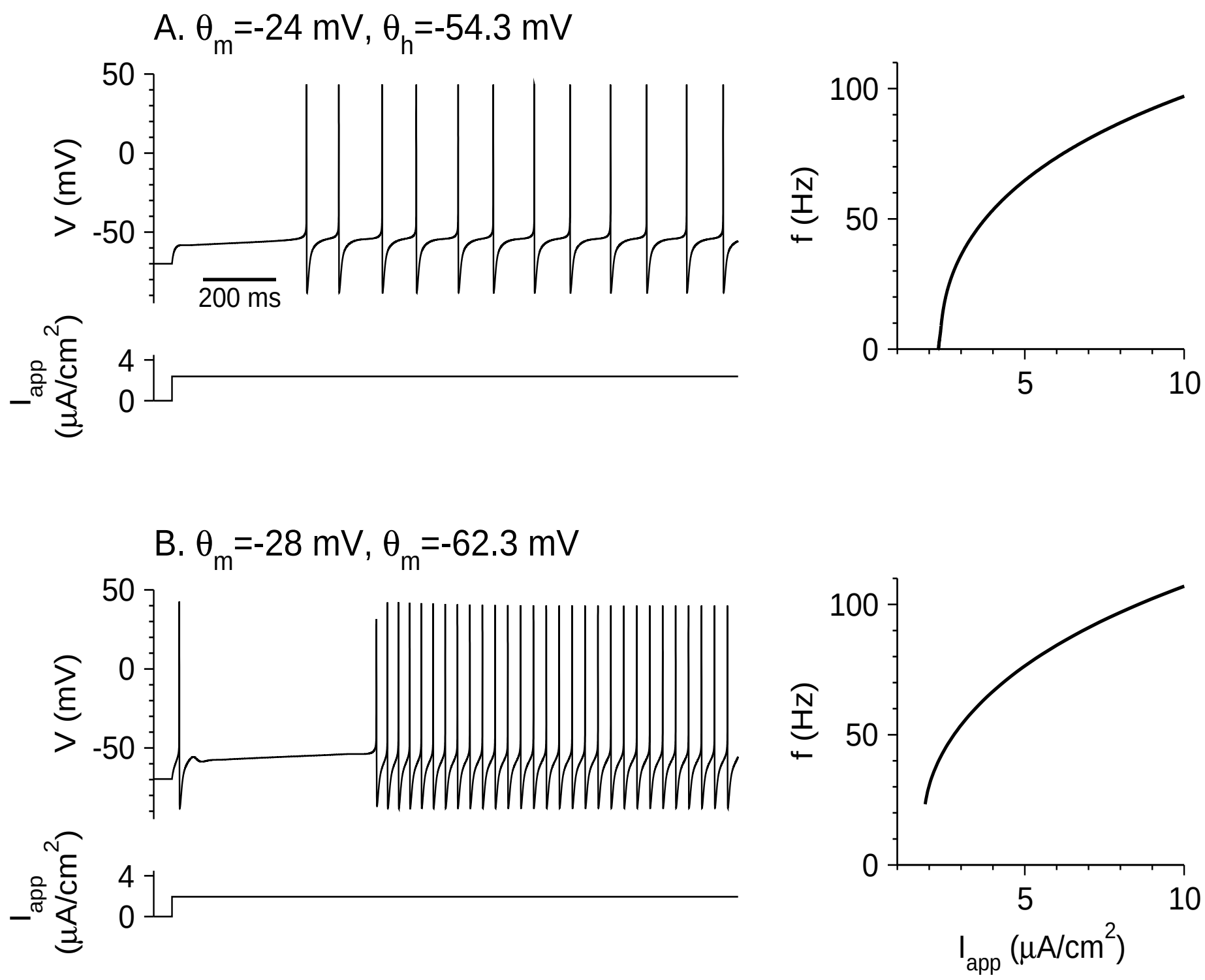

Figure S2. Response of the model neuron to noiseless current steps for modified θ_h values.

A. Parameters: $\theta_\mathrm{m} = -24\,\mathrm{mV}$, $\theta_\mathrm{h} = -54.3\,\mathrm{mV}$, $g_\mathrm{d} = 0.39\,\mathrm{mS/cm^2}$. Left: voltage trace for $I_\mathrm{app} = 2.38\,\mu\mathrm{A/cm^2}$. Right: the steady-state f-I_app curve of the neuron. The average firing frequency goes to zero at firing threshold. B. Parameters: $\theta_\mathrm{m} = -28\,\mathrm{mV}$, $\theta_\mathrm{h} = -62.3\,\mathrm{mV}$, $g_\mathrm{d} = 0.39\,\mathrm{mS/cm^2}$. Left: voltage trace for $I_\mathrm{app} = 1.95\,\mu\mathrm{A/cm^2}$. Right: the steady-state f-I_app curve is discontinuous at the current threshold. The minimal frequency is 23.3 Hz.
